# Near-Field Electrospun Piezoelectric Fibers as Sound-Sensing Elements

**DOI:** 10.3390/polym10070692

**Published:** 2018-06-21

**Authors:** Tien Hsi Lee, Chun Yu Chen, Chen Yu Tsai, Yiin Kuen Fuh

**Affiliations:** 1 Department of Mechanical Engineering, National Central University, No. 300, Jhongda Rd., Jhongli District, Taoyuan City 32001, Taiwan; benlee@ncu.edu.tw (T.H.L.); su3cl3x87@yahoo.com.tw (C.Y.C.); jason82621@gmail.com (C.Y.T.); 2 Institute of Energy Engineering, National Central University, Taoyuan City 32001, Taiwan

**Keywords:** near-field, electrospun, piezoelectric fibers

## Abstract

A novel integration of three-dimensional (3D) architectures of near-field electrospun polyvinylidene fluoride (PVDF) nano-micro fibers (NMFs) is applied to an intelligent self-powered sound-sensing element (ISSE). Using 3D architecture with greatly enhanced piezoelectric output, the sound wave energy can be harvested under a sound pressure of 120+ dB SPL of electrical signal about 0.25 V. Furthermore, the simple throat vibrations such as hum, cough and swallow with different intensity or frequency can be distinguishably detected. Finally, the developed ultrathin ISSE of near-field electrospun piezoelectric fibers has the advantage of direct—write fabrication on highly flexible substrates and low cost. The proposed technique demonstrates the advancement of existing electrospinning technologies in new practical applications of sensing purposes such as voice control, wearable electronics, implantable human wireless technology.

## 1. Introduction

**Figure 1:**
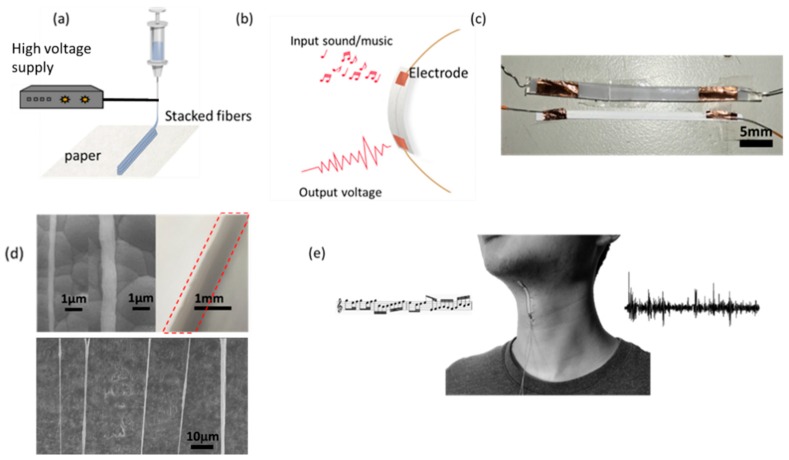
Schematic illustration of the fabrication process and the morphology of ISSE. (**a**) One-step fabrication process of ISSE. Nano/micro PVDF fibers were directly deposited on the printing paper by using near-field electrospinning (NFES). (**b**) ISSE has the ability of detecting sound in one device. (**c**) The optical photo of sound sensing element. (**d**) The morphology of two nano/micro fibers (**top**) and multiple aligned fibers (**bottom**) as produced at 1.5 kV. Due to the spinnability of NFES, the fiber diameters were typically in the range of 500 nm to 3 μm. (**e**) The artificial throat can detect the movement of throat and music sound, respectively. The controllability of the stacked ~100 layer electrospun PVDF fibers wall structure is demonstrated by a variety of NFES processing conditions such as x–y translational motion stage speed, PVDF weight-volume, and spinner-to-collector distance.

As the increasing needs for the extreme integration of micro/nanodevices with versatile functionalities, the multifunctional micro/nanosystems are highly pursuit for transduction of energy in the fields of environmental, biomechanical and human–machine interfacing [1,2,3,4,5]. In particular, it is of great significance to develop sound sensing devices or acoustic transducers within the human hearing range for the benefit of human being [6]. One of the key thrust areas lie in the nanomaterials and nanotechnologies for the unique novelty and new mechanisms. For example, grapheme [7,8,9], carbon nanotube [10,11], zinc oxide (ZnO) nanowires (NWs) [12,13], lead zirconate titanate (PZT) NWs [14], PZT materials [15,16,17], PVDF fibers [18,19], silver nanowires [20] and indium tin oxide [21] have promised a great avenue of innovation in the wearable devices with the required features of flexible and self-powered ability. Concerning the development of self-powered systems, triboelectric nanogenerators (TENGs) were rigorously rekindled to harvest ambient mechanical energy [22], to promote the self-powered human-machine interfacing [23] and in-situ monitoring of the motion vector sensor [24]. Another category of self-powered systems is the recently developed near-flied electrospun (NFES) polyvinylidene fluoride (PVDF) fibers [25,26,27]. The direct-write NFES is capable of producing highly aligned piezoelectric PVDF nanofibers with in situ mechanical stretching and electrical poling (the electric field is ~10^7^ V/m) such that the nanofiber crystal of randomly oriented dipoles is transformed into polar phase, eliminating the need for further post-poling process. The direct-write technique by means of NFES to produce and place piezoelectric PVDF nanofibers on working substrates is shown in Figure 1a). The strong electric fields (greater than 107 V/m) and stretching forces from the electrospinning process naturally align dipoles in the nanofiber crystal such that the nonpolar R phase (random orientation of dipoles) is transformed into polar phase, determining the polarity of the electrospun nanofiber. Recent advancement of PVDF electrospun fibers with near-field electrospinning (NFES) setup can produce piezoelectric response of electrical outputs 5–30 mV and 0.5–3 nA [27] and furthermore, scaing up of 500 microfibers were reported to be capable of producing a peak output voltage of ~1.7 V and the current of ~300 nA [28]. In this study, the 3D piezoelectric PVDF fibers were sequentially constructed on the paper substrate to fabricate the highly flexible and structurally durable piezoelectric sound-sensing elements with a simple processing and low-cost strategy. The ultrathin intelligent self-powered sound-sensing elements can tightly cohere on the human throat and loud speaker, can not only detected the vibration cause by sound but also detect the human motion without external energy source.

## 2. Experimental Results

### 2.1. Experimental Design

The NFES manufacturing process and the structure of the intelligent and self-powered sound-sensing elements (ISSE) is schematically illustrated in Figure 1a,b. ISSE can be repeatedly direct-write, in-situ poled via the 3D NFES electrospinning technique on the paper substrate. Owing to an enhanced electrical charge transfer as resulted from the paper substrate (Double A, Hong Kong, China, HYL080) of the infiltrated solvent between the deposited fibers and the conductive ground plate, the formation of 3D fibrous network within the paper can be vertically stacked [5] in the compact and ultrathin package (2.5 × 50 × 2 mm^3^, 2.5 mm in width). As illustrated in Figure 1b, the ISSE has the integrated functions of detecting sounds and movements. When sounds or pressure are applied on the device, for example the vibration of throat, the deformation-induced strain will cause the accumulation of uni-poled dipole moment and piezoelectric potential across the length-wise of the stacked electrospun fibers such that the output the voltage/current can be generated as a result of piezoelectric principle. All authors agreed on the ethics approval and consent to participate.

### 2.2. Materials and Methods

The optical photo of ISSE is shown in Figure 1c Regarding the NFES solution preparation, 16 wt % PVDF, solvent (DMF: acetone with 1:1 weight ratio), 4 wt % fluoro-surfactant (ALDRICH, Saint Louis, MO, USA, Capstone FS-66), 3D structural integrity can be ensured by properly selecting the balance between the electrostatic, capillary and evaporative forces [29]. For material characterization the spectroscopic evidence of X-ray diffraction (XRD) and Fourier transform infrared spectroscopy (FTIR) are collected and presented in Supplementary Information Figures S1 and S2, respectively. Figure 1d shows SEM photograph of two typical NFES electrospun PVDF fibers constructed on a paper substrate with sputtered gold electrode. The general NFES processing process parameters adopted in this paper are as follows: applied voltage at 1.6 kV, motion speed of 60 mm/s, and initial spinneret-to-collector distance of 2 mm. Due to the unique characteristics of NFES (solution spinnability and the continuous deposition are the two limiting factors), the electrospun fiber diameters were typically in the range of 500 nm to 3 μm. The mean diameter calculations of the fibers are performed by collecting the SEM images of ~30 fibers such that the average diameters can be deduced. The detail processing window of stacked fibers will be discussed in next session. Figure 1e shows the energy harvesting potential of scavenging biomechanical human motion such as cough, hum or scream will cause the vibration of throat cords and can be detected by the ISSE as the artificial throat accordingly. The working principle of the ISSE lies critically in the aligned dipoles y since electrical potentials can only be generated under axial strain by bending the bottom plastic substrate. Details on the working principle of ISSE can be found in Supplementary Information Figure S3.

### 2.3. Experimental Results

**Figure 2:**
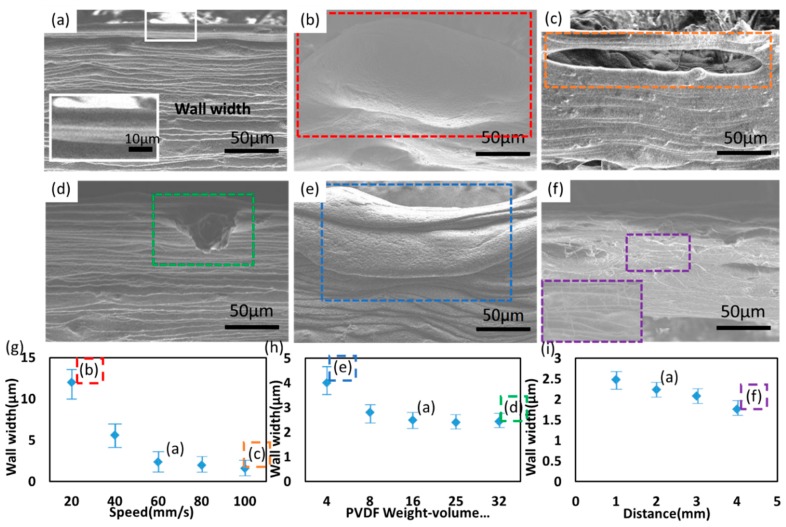
(**a**) The morphology of 3D electrospun PVDF fibers with the spinneret size of 27 G, applied voltage at 1.6 kV, 16 wt % PVDF solution, 60 mm/s x-y stage speed and 2 mm spinneret-to-collector distance under SEM. The above processing conditions are selected as the basis. The inset shows a magnified view. SEM images showing the morphology of 3D electrospun PVDF fibers with respect to (**b**) 20 mm/s x-y stage speed (**c**) 100 mm/s x-y stage speed (**d**) 32 wt % PVDF solution (**e**) 8 wt % PVDF solution (**f**) 4 mm spinneret-to-collector distance. Plots showing the dependence of wall width with respect to (**g**) PVDF weight-volume percentage, (**h**) motion speed of the x-y stage, (**f**) spinneret-to-collector distance. The experimental data are the collection of ten samples, with the average values and error bars showing the distribution. The insets of red/orange/green/blue/purple dotted rectangle in (**g**–**i**) correspond to the NFES processing parameters in (**b**–**f**).

Figure 2a shows a free-standing 3D NFES PVDF fibers wall as produced by the standard electrospinning process includes: motion stage speed of 60 mm/s, applied voltage at 1.6 kV, spinneret size of 27 G, spinneret-to-collector distance of 2 mm, and solution concentration of 16 wt %. Only one parameter is altered for each graph. Figure 2b,c show the SEM morphology of PVDF fibers as electrospun at slower and faster x-y stage speed of 20 and 100 mm/s, respectively. Both speeds create the defected fibers of aggregation (Figure 2b), split (Figure 2c). In addition, using a dense concentration of 32 wt % PVDF solution, fiber agglomeration can be observed (Figure 2d). Whereas the lean concentration of 4 wt % PVDF solution structurally stable stacked fibers cannot be sustained (Figure 2e). In addition, increasing the spinneret-to-collector distance to 4 mm will incur the randomly distributed ultrafine fibers (10–50 nm), similar to conventional electrospinning process (Figure 2f). Figure 2g–i plots showing the dependence of wall width of NFES fibers with respect to (Figure 2g) PVDF Weight-volume percentage, (Figure 2h) motion speed of the x-y stage, (Figure 2i) spinneret-to-collector distance. In general, the width of the 3D stacked fibers is typically larger to the counterpart of a single fiber due to the effect of stacking instability in the non-vacuum environment. For example, the width tunability range can be as small as 0.5 ± 0.33 to 14 ± 0.8 μm by adjusting the various processing parameter of x-y stage speed. For example, the speed of the motion stage in Figure 2g are found to be of crucial importance in generating the extreme case (14 ± 0.8 μm, corresponds to the SEM in Figure 2b). Higher motion stage speed can be regarded as higher mechanical drawing force and thus cause the reduction of wall width, which is in agreement with previous study [29]. On the contrary, the less-optimal conditions can result in the defected fibers of aggregation due to poor solvent evaporation (Figure 2b), split due to poor spinnability (Figure 2c), agglomeration due to insufficient dissolution of the PVDF solution (Figure 2d), deformed stacked fibers due to lean PVDF solution (Figure 2e), and even the formation of randomly distributed ultrafine fibers (10–50 nm) on top of NFES fibers (Figure 2f). All above defects will inevitably resulting in failure of fiber stacking. Therefore, a continuously deposited 3D NFES process should always exclude the processing parameters as illustrated in Figure 2b–f. Conversely, a lower motion stage speed results in defects of the form of piling up, buckling, and random coiling of fibers [29,30].

**Figure 3:**
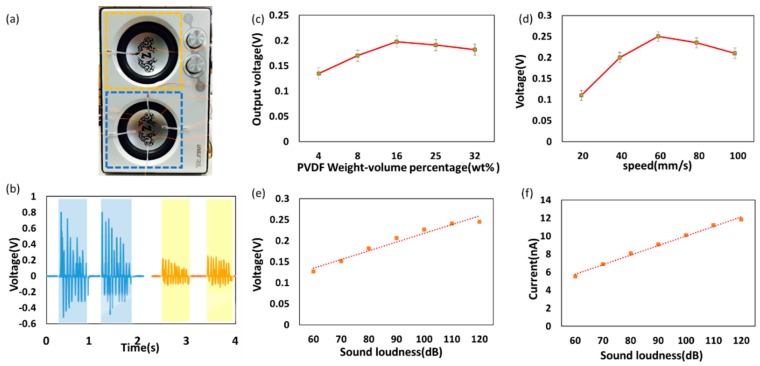
The performance of sensing sound. The ISSE is clamped on a loudspeaker to test the sound-sensing capability as illustrated in (**a**) optical photo. (**b**) The relative voltage output detected by four ISSE (blue dotted rectangle) and one ISSE (blue dotted rectangle). (**c**) The relative voltage output as a function of the PVDF solution concentration from 8% to 32%. The x-y stage speed is fixed at 60 mm/s. (**d**) The relative voltage output as a function of the x-y stage speed from 20 to 100 mm/s. The PVDF solution concentration is fixed at 16%. The occurrence of voltage peak coincides with the NFES parameters of 16% PVDF solution and at 60 mm/s motion speed, which is the defect-free morphology and higher quality. The recorded (**e**) relative voltage output and (**f**) current output. Both electrical signals increase with the increase of the sound loudness of the loudspeaker.

Electricity-generating performance of proposed ISSE at various sound loudness and NFES processing conditions under biomechanical human voice of hum was initially recorded and broadcasted by loudspeaker (T. C. Star, tcs3428) in Figure 3. The sound pressure can be tuned by adjusting the output of loudspeaker. ISSE was adhered to the loudspeaker membrane tightly as schematically shown in optical photo Figure 3a. The ISSE shows a highly addition ability in Figure 3b relative voltage output has detected by four ISSE (blue dotted rectangle) and one ISSE (yellow dotted rectangle). In order to investigate the performance of ISSE under different NFES processing conditions and the resulted morphologies as well as defects, the sample fabrication in previous experiment has been used to verification. As shown in Figure 3c,d, ISSE has higher output at PVDF weight-volume percentage 16% and at processing speed 60 mm/s and the morphology of PVDF fibers sample is corresponding to Figure 2a. Moreover, if the suboptimal conditions are chosen such as excessive the spinning fibers due to slow motion stage speed or high concentration of PVDF weight-volume percentage. The output voltage will be drop, which can be attributed to the defect and fiber width of the PVDF fibers wall as shown in Figure 2b–e. The reasons for the optimal performance of the electrospun ISSE can be attributed primarily to the defect-free morphology and better quality. As shown in Figure 3e, the output voltage of ISSE is linearly increases from 0.13 to 0.23 V with the input of sound loudness from 60 to 120 dB and same linear phenomenon can be observed between output current and input of sound loudness as shown in Figure 3f. Similar trend can be found on the previously reported paper-based acoustic energy harvesting triboelectric generator such that the dependence of the electrical output was a direct proportional function with the incident sound pressures [30].

Furthermore, the forward and reverse connections measurements of the NFES electrospun NMFs have been validated and presented in Supplementary information Figure S4. Polarity test is crucial to validate polarity switched effect of the authentic piezoelectric responses and exclusion of any background or triboelectric signals. Furthermore, Supplementary Information Figure S5 shows the principle of superposition of the fabricated ISSE devices in serial connection modes. To be equivalently characterized as a self-powered sensor in a resistance matching setup, Supplementary information Figure S6 shows the experimentally measured output voltage and power against external load resistance. The experiments show that the load voltage follows a monotonic increasing trend as the load resistance increases while the power output reaches the optimized output power of 1.2 nW at matched resistance of 10 MΩ. In addition, the stability of the flexible paper-based self-powered sensor is an essential factor to ensure its long-term durability during the stages of applications. Three days continuous output voltage and output current of the ISSE operating at 2 Hz as shown in Supplementary information Figure S7. From the experimental results, the fabricated ISSE exhibits stable robustness of peak output voltage8, indicating the durable power generation of the ISSE.

**Figure 4:**
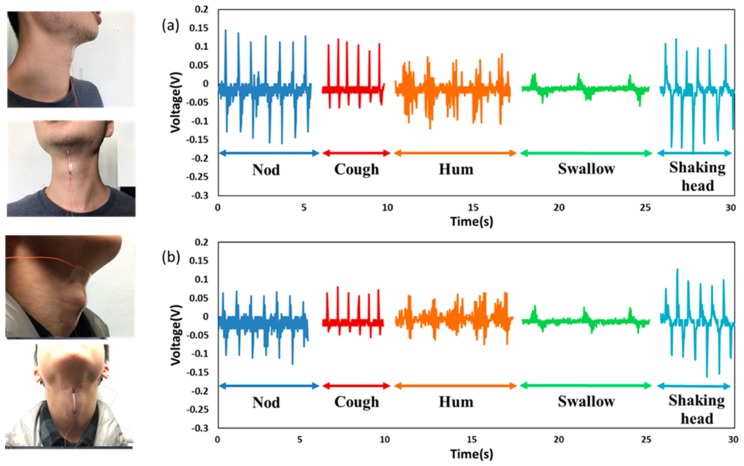
Responses towards different kinds of throat vibrations. (**a**) The ISSE’s output voltage with various throat movements (nod, coughs, hums, swallow and shaking head) of the test subject (A). (**b**) Same test as performed by subject (B).

After identifying the potential for harvesting the acoustic energy, ISSE is used to detect the vibration of throat cords. As shown in Figure 4, the first tester makes several successive different actions which are coughs, hums, shaking head, swallowing and nod action. The repeatability of the detection is excellent according to the several-time successive testing. Besides, the swallowing, nod and shaking head can cause the muscle and Adam’s apple movement, which can also result in the deformation of the ISSE, and initiate piezoelectric reaction. Fortunately, the waveforms of these kinds of muscle movements also have recognizable characteristics. Different movement has its unique characteristic waveform as shown in Figure 4a; thus, we can get the useful waveforms by relying on the pattern recognition and machine learning. The interference by some other activities can be recognized and eliminated by training many times in advance. Then, the second tester makes the same actions which the first tester made as shown in Figure 4b.

**Figure 5:**
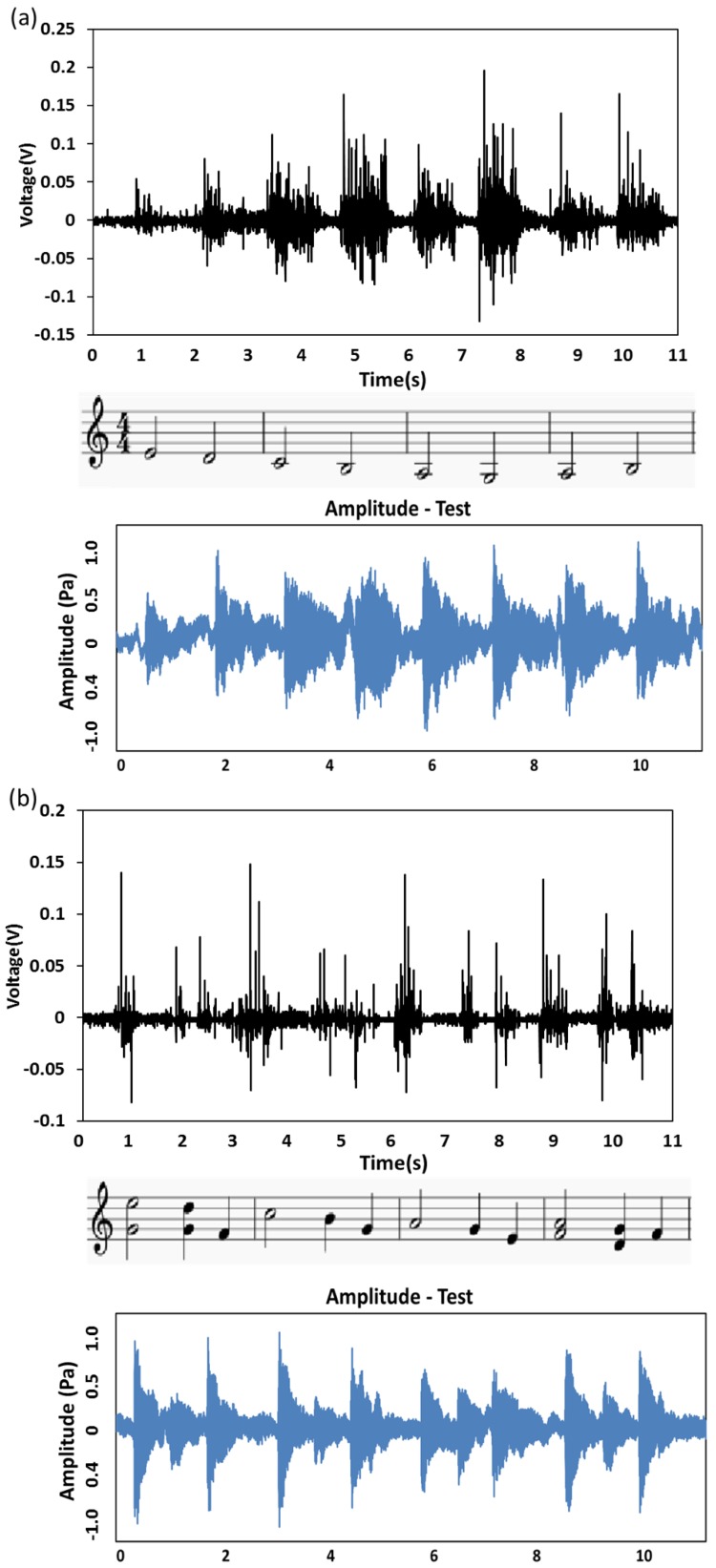

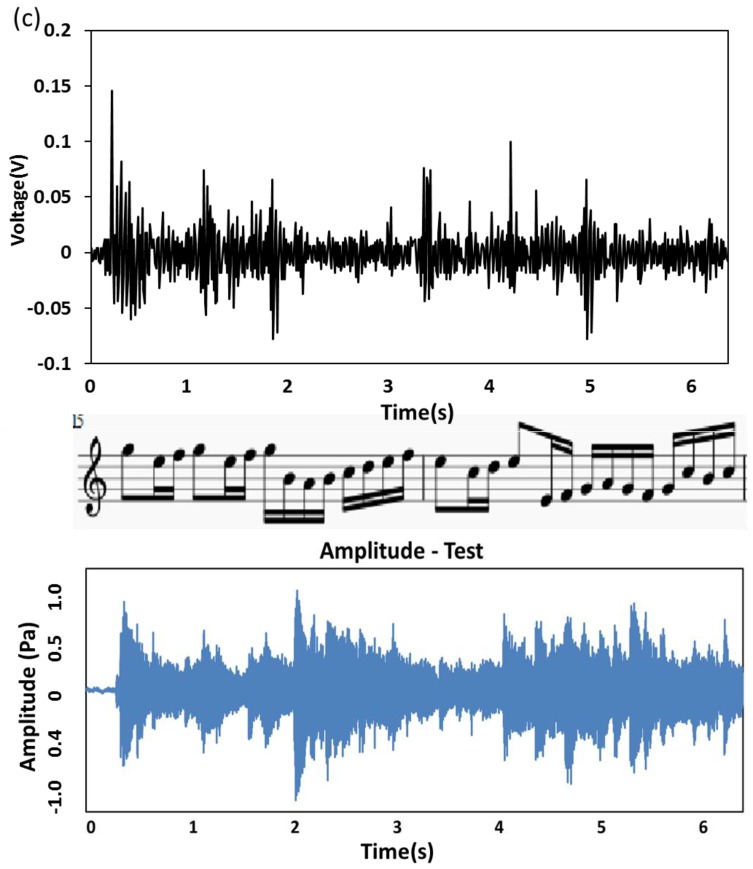
The ISSE showing the capability of voice recognition. The ISSE is placed on the top of the loudspeaker and recorded responses towards different scores of Canon-in-D (**a**) 1–4 measures of Canon-in-D (**b**) 9–12 measures of Canon-in-D (**c**) 29 and 30 measures of Canon-in-D. The black image in the upper row indicates the voltage output responses towards different parts of Canon-in-D as measured by ISSE in a self-powered manner and the staff notation in the middle row is corresponding to the three scores of Canon-in-D. The blue image in the bottom row indicates the amplitude profiles of the original audios as measured from a complete acoustic acquisition system and microphone. Relative voltage output as measured by ISSE corresponds to nearly synchronous response to the meter-measured profiles of the original audios.

ISSE can be functionally working as a sound detector in the self-powered fashion, schematically presented in Figure 5 The ISSE has excellent responses when detecting sound and the sensitivity is high enough to detect sound amplitude produced by a loudspeaker. The ISSE is fixed on the loudspeaker membrane by glue and adhesive plaster. The audio tests with three sections of music audios of Cannon in D are performed. Figure 5a shows the 1–4 measures of Canon-in-D, similarly as shown in Figure 5b the 9–12 measures of Canon-in-D and Figure 5c the 29 and 30 measures of Canon-in-D. The black image in the upper row indicates the voltage output responses towards different parts of Canon-in-D as measured by ISSE in a self-powered manner and the staff notation in the middle row is corresponding to the three scores of Canon-in-D. The blue image in the bottom row indicates the sound amplitude profiles of the original audios as measured from a testing apparatus consists of a complete acoustic acquisition system NI 9234 (Analog Input Modules (IEPE) 4-Ch). An acoustic microphone (PCB Group, Inc., New York, NY, USA, 130E21) setup was employed as the device for receiving sound amplitude and measuring different acoustical harmonics. Measured voltage outputs show almost synchronous response to profiles of the original audios when the loudspeaker plays the audio. Especially, three sections of Cannon in D show almost identical resemblance from the corresponding images of the sound amplitude and voltage output profiles, only forte and piano change. Compared with some other nanomaterial-based acoustic detectors, our device demonstrates superior predominance in voice recognition because of its excellent repeatability and reliability.

## 3. Conclusions

In summary, a direct-write, self-aligned and one-step fabricated sound sensing element based on PVDF fibers has been developed and functions in the self-powered manner. The ISSE realizes the functional integration of piezoelectric materials as sound detectors to capture the bio-mechanical vibration of human motion such as throat cords. This developed process enables orderly fiber-by-fiber stacking to construct 3D structures in a controllable and prescribed pattern. The distinctive in situ electrical poling and mechanical stretching in NFES process to deposit one row of 200 PVDF NMFs can create a peak output voltage of ~132 mV and a current reaching 8 nA at the optimized resistor matching of 10 MΩ. It can be demonstrated that distinguishable characteristics of nod, head shaking, cough, hum and swallow can be clearly differentiated. In addition, the musical scores of unique waveforms as played in Cannon in D are also vividly captured by the ISSE with different tones and volumes such that the voice recognition capability is demonstrated. Therefore, this 3D electrospinning nanofabrication technique has the potential in constructing 3D structures for wearable electronics as the voice control and recognition devices.

## Supplementary Materials

The following are available online at http://www.mdpi.com/2073-4360/10/7/692/s1.

## References

[1] Ko H.C., Stoykovich M.P., Song J., Malyarchuk V., Choi W.M., Yu C.J., Geddes J.B., Xiao J., Wang S., Huang Y. (2008). A hemispherical electronic eye camera based on compressible silicon optoelectronics. Nature.

[2] Keplinger C., Sun J.Y., Foo C.C., Rothemund P., Whitesides G.M., Suo Z. (2013). Stretchable, transparent, ionic conductors. Science.

[3] Dvir T., Timko B.P., Brigham M.D., Naik S.R., Karajanagi S.S., Levy O., Jin H., Parker K.K., Langer R., Kohane D.S. (2011). Nanowired three-dimensional cardiac patches. Nat. Nanotechnol..

[4] Wu W., Wen X., Wang Z.L. (2013). Taxel-addressable matrix of vertical-nanowire piezotronic transistors for active and adaptive tactile imaging. Science.

[5] Sekitani T., Yokota T., Zschieschang U., Klauk H., Bauer S., Takeuchi K., Takamiya M., Sakurai T., Someya T. (2009). Organic nonvolatile memory transistors for flexible sensor arrays. Science.

[6] Tao L., Tian H., Liu Y., Ju Z., Pang Y., Chen Y., Wang D., Tian X., Yan J., Deng N. (2017). An intelligent artificial throat with sound-sensing ability based on laser induced grapheme. Nat. Commun..

[7] Tian H., Ren T.L., Xie D., Wang Y.F., Zhou C.J., Feng T.T., Fu D., Yang Y., Peng P.G., Wang L.G. (2011). Graphene-on-paper sound source devices. ACS Nano.

[8] Suk J.W., Kirk K., Hao Y., Hall N.A., Ruoff R.S. (2012). Thermoacoustic sound generation from monolayer graphene for transparent and flexible sound sources. Adv. Mater..

[9] Tian H., Li C., Mohammad M.A., Cui Y.L., Mi W.T., Yang Y., Xie D., Ren T.L. (2014). Graphene earphones: Entertainment for both humans and animals. ACS Nano.

[10] Brown L.F., Carlson D.L. (1989). Ultrasound transducer models for piezoelectric polymer films. IEEE Trans. Ultrason. Ferroelectr. Freq. Control.

[11] Mason B.J., Chang S., Chen J., Cronin S.B., Bushmaker A.W. (2015). Thermoacoustic transduction in individual suspended carbon nanotubes. ACS Nano.

[12] Zhu G., Yang R., Wang S., Wang Z.L. (2010). Flexible high-output nanogenerator based on lateral ZnO nanowire array. Nano Lett..

[13] Lee M., Bae J., Lee J., Lee C.S., Hong S., Wang Z.L. (2011). Self-powered environmental sensor system driven by nanogenerators. Energy Environ. Sci..

[14] Chen X., Xu S., Yao N., Shi Y. (2010). 1.6 V nanogenerator for mechanical energy harvesting using PZT nanofibers. Nano Lett..

[15] Gu L., Cui N., Cheng L., Xu Q., Bai S., Yuan M., Wu W., Liu J., Zhao Y., Ma F. (2012). Flexible fiber nanogenerator with 209 V output voltage directly powers a light-emitting diode. Nano Lett..

[16] Wu W.W., Bai S., Yuan M.M., Qin Y., Wang Z.L., Jing T. (2012). Lead zirconate titanate nanowire textile nanogenerator for wearable energy-harvesting and self-powered devices. ACS Nano.

[17] Lee J.H., Lee K.Y., Kumar B., Tien N.T., Lee N.E., Kim S.W. (2013). Highly sensitive stretchable transparent piezoelectric nanogenerators. Energy Environ. Sci..

[18] Luo G., Teh K.S., Liu Y., Zang X., Wen Z., Lin L. (2015). Direct-write, self-aligned electrospinning on paper for controllable fabrication of three-dimensional structures. ACS Appl. Mater. Interfaces.

[19] Jiang Y., Gong L., Hu X., Zhao Y., Chen H., Feng L., Zhang D. (2018). Aligned P(VDF-TrFE) Nanofibers for Enhanced Piezoelectric Directional Strain Sensing. Polymers.

[20] Tian H., Xie D., Yang Y., Ren T.L., Lin Y.X., Chen Y., Wang Y.F., Zhou C.J., Peng P.G., Wang L.G. (2011). Flexible, ultrathin, and transparent sound-emitting devices using silver nanowires film. Appl. Phys. Lett..

[21] Tian H., Xie D., Yang Y., Ren T.L., Wang Y.F., Zhou C.J., Peng P.G., Wang L.G., Liu L.T. (2011). Transparent, flexible, ultrathin sound source devices using indium tin oxide films. Appl. Phys. Lett..

[22] Yang P.K., Lin Z.H., Pradel K.C., Lin L., Li X., Wen X., He J.H., Wang Z.L. (2015). Paper-based origami triboelectric nanogenerators and self-powered pressure sensors. ACS Nano.

[23] Chen J., Zhu G., Yang J., Jing Q., Bai P., Yang W., Qi X., Su Y., Wang Z.L. (2015). Personalized keystroke dynamics for self-powered human-machine interfacing. ACS Nano.

[24] Jing Q., Xie Y., Zhu G., Han R.P.S., Wang Z.L. (2015). Self-powered thin-film motion vector sensor. Nature.

[25] Sun D., Chang C., Li S., Lin L. (2006). Near-field Electrospinning. Nano Lett..

[26] Chang C., Limkrailassiri K., Lin L. (2008). Continuous Near-field Electrospinning for Large Area Deposition of Orderly Nanofiber Patterns. Appl. Phys. Lett..

[27] Chang C., Tran V.H., Wang J., Fuh Y.K., Lin L. (2010). Direct-write piezoelectric polymeric nanogenerator with high energy conversion efficiency. Nano Lett..

[28] Fuh Y.K., Chen S.Y., Ye J.C. (2013). Massively parallel aligned microfibers-based harvester deposited via in situ, oriented poled near-field electrospinning. Appl. Phys. Lett..

[29] Fuh Y.K., Li S.C., Chen C.Y. (2017). Piezoelectrically and triboelectrically hybridized self-powered sensor with applications to smart window and human motion detection. APL Mater..

[30] Fan X., Chen J., Yang J., Bai P., Li Z., Wang Z. (2015). Ultrathin, Rollable, Paper-Based Triboelectric Nanogenerator for Acoustic Energy Harvesting and Self-Powered Sound Recording. ACS Nano.

