# Supplementary material for: Near-Field Electrospun Piezoelectric Fibers as Sound-Sensing Elements
Source: Polymers (Basel). 2018 Jun 21;10(7):692. doi: 10.3390/polym10070692 (PMC6403917; doi:10.3390/polym10070692)
Supplement: Supplementary file 1 [file polymers-10-00692-s001.pdf]

polymers

MDPI

Supplementary information

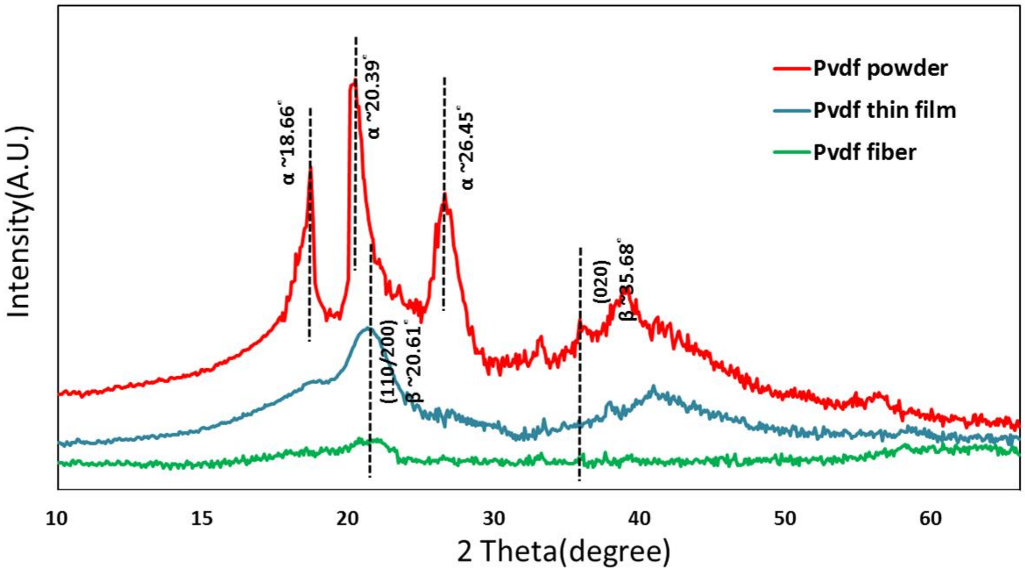

Figure S1. XRD patterns of original PVDF powder (red line), NFES PVDF fiber (green line) and conventional electrospinning PVDF thin film (blue line). XRD measurement results show two obvious diffraction peaks at 2θ = 18.66°, 20.39° and a weaker peak at 2θ = 26.45° corresponding to 020, 110 and 021 reflections of the α-phase (monoclinic) crystal, respectively. The NFES PVDF fiber (green line) and conventional electrospinning PVDF thin film (blue line) are both observed a very strong diffraction peak at 2θ = 20.61° and a weak peak at 2θ = 35.68° corresponding to 110/200 and 020 reflections of the β-phase (orthorhombic) crystal, respectively.

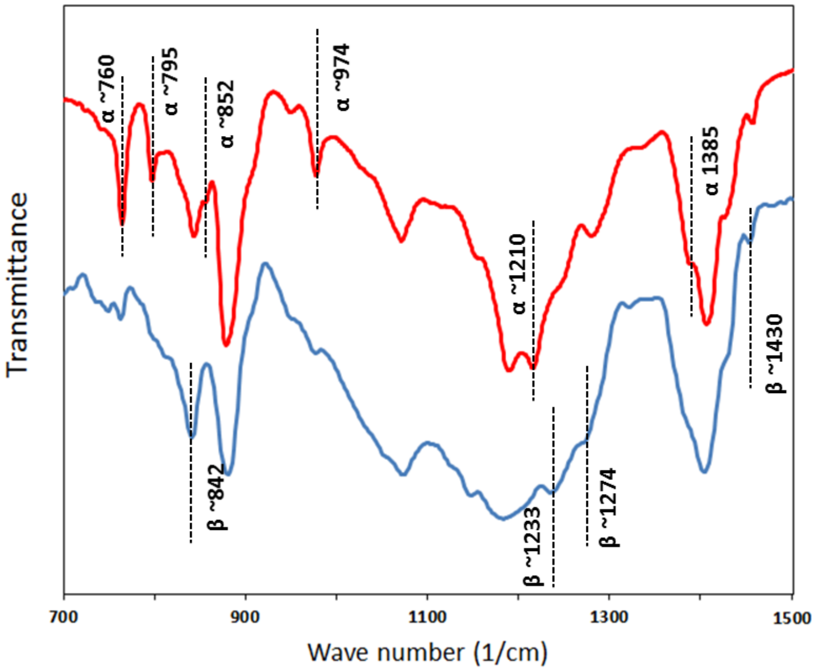

Figure S2. FTIR spectra of the PVDF powder and electrospinning PVDF fibers. The characteristic transmittance bands of the β-phase are measured at 842, 1233, 1274, and 1430 cm^{-1} in PVDF fiber (blue line). On the other hand, the characteristic transmittance bands of the non-polar α-phase were observed in PVDF powder (red line) at 760, 795, 852, 974, 1210, and 1385 cm^{-1}.

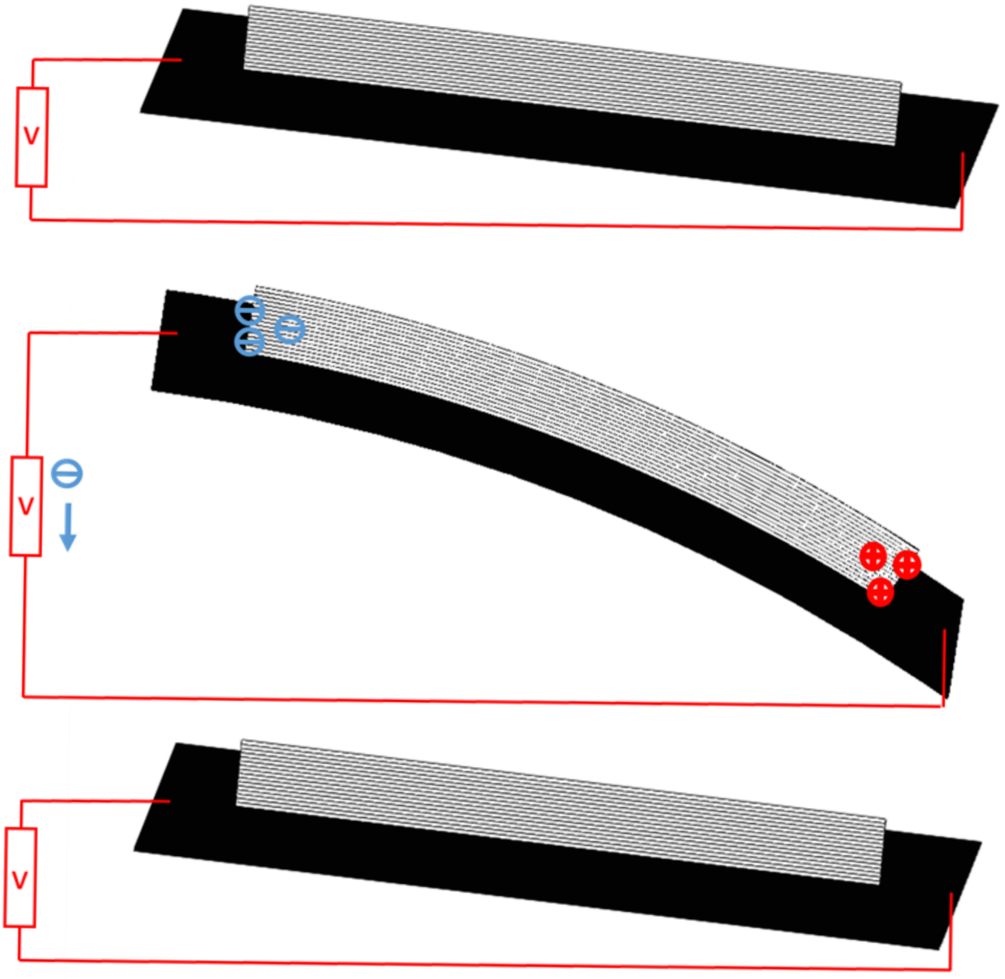

Figure S3. ISSE working principle. Working principle of the voltage and current scaling-up superposition for the electrospun NMFs are integrated as one large array energy harvesting device. As mechanical deformation was induced on the substrate, tensile strain and a corresponding piezoelectric potential in the NMFs can be created, where the "±" signs indicate the polarity of the local piezoelectric potential created in situ inside the NMFs.

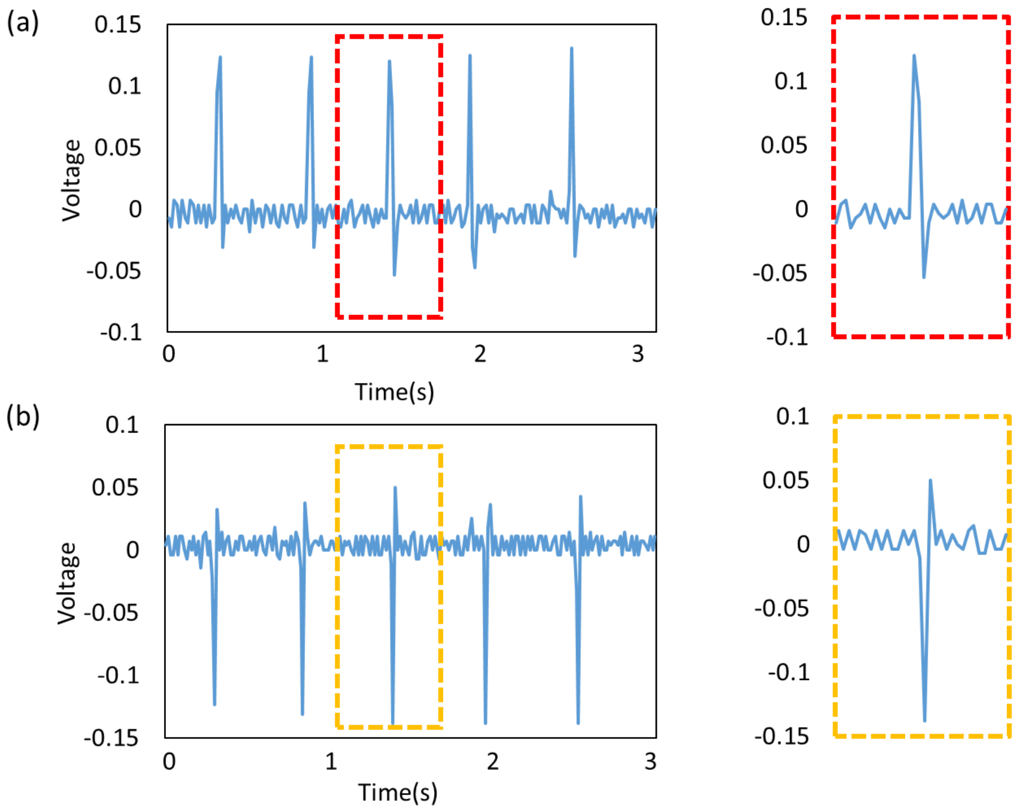

Figure S4. Validated tests of polarity as implemented via forward and reverse connections measurements. The shape of output signal changed as switching the measurement polarity. (a) The peak voltage and currents generated by the ISSE of about 0.13V were obtained in the forward connection. (b) The output voltages and currents generated by the ISSE of about 0.14V in the reverse connection. All the tests were operating at strain of 0.5% and ~2Hz.

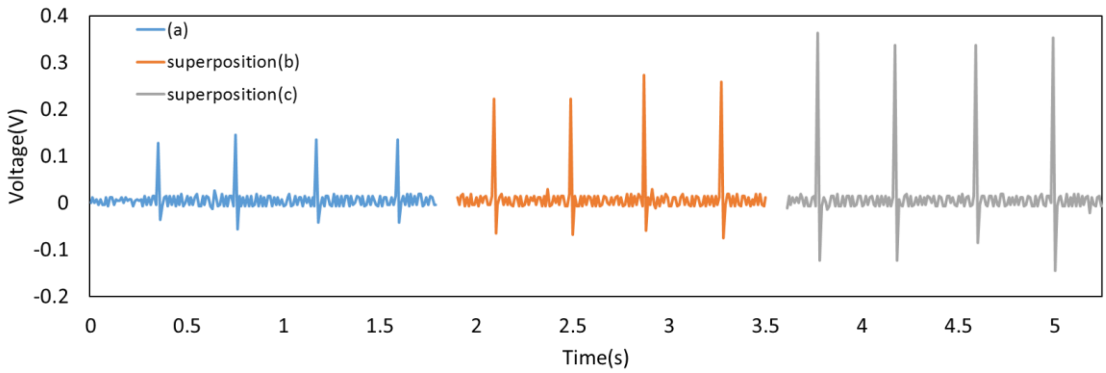

Figure S5. Validated tests of superposition as implemented via series connections measurements. Output voltages of (a) one ISSE subject to continuous stretch and release. Output voltages constructively add when (b) two ISSEs (c) three ISSEs are in serial connection. All data are measured when the two WSSs are operated at the same

humans hum sound played by loudspeaker, and frequency (~2Hz).

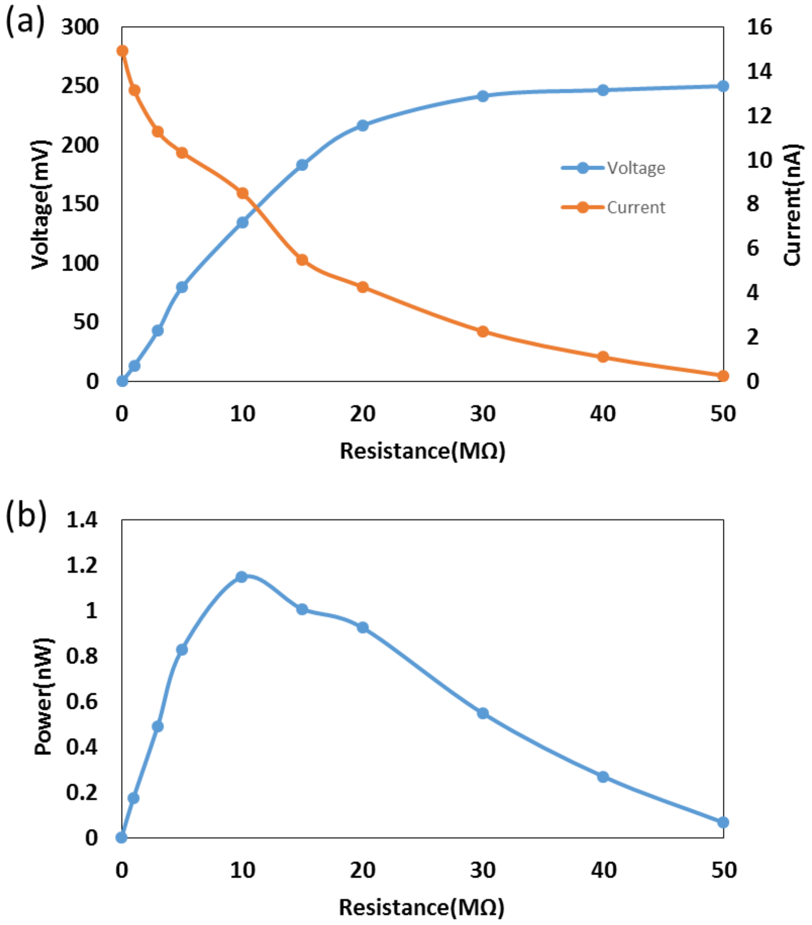

Figure S6. Impedance matching tests of ISSE. The ISSE could be modeled with different load resistors for the impedance matching test. Repeatedly pressing the ISSE at 2 Hz created a maximum peak voltage and power of 132 mV and 1.18nW, from the ISSE with an external load resistance of 10MΩ.

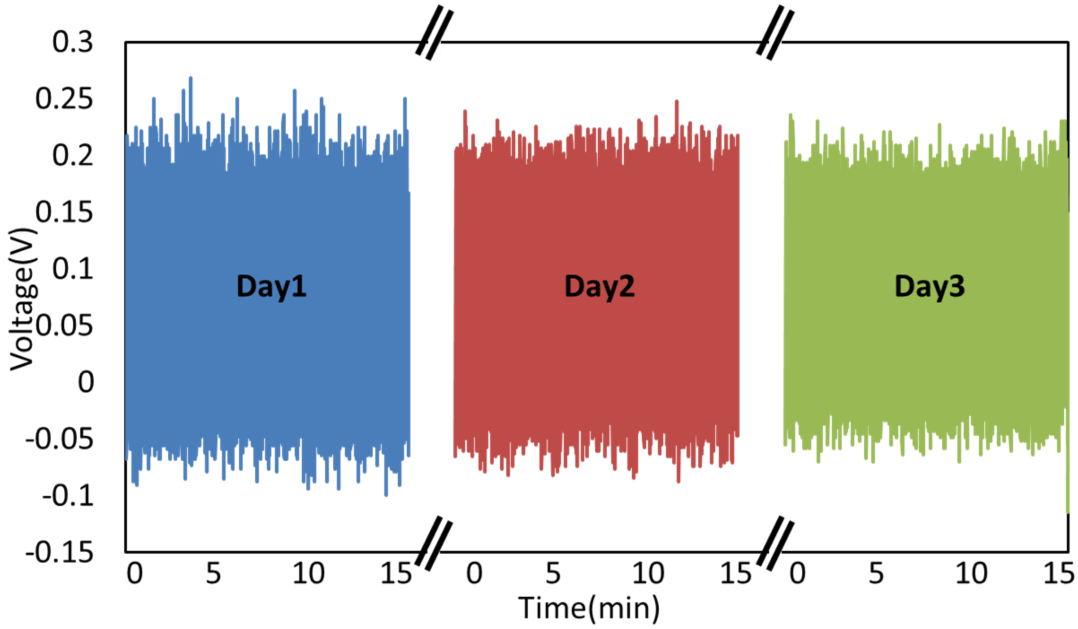

Figure S7. Long-term stability tests. Output voltage of a PVDF PG was put under 2Hz of continuous cycles of a humans hum sound played by loudspeaker for 3 days, demonstrating the stability of the ISSE. The ISSE was continuously run each day for 15min.
